# Functional Corticomuscular Signal Coupling Is Weakened during Voluntary Motor Action in Cancer-Related Fatigue

**DOI:** 10.1155/2019/2490750

**Published:** 2019-06-26

**Authors:** Changhao Jiang, Qi Yang, Tingting Chen, Vlodek Siemionow, Vinoth K. Ranganathan, Alice F. Yan, Guang H. Yue

**Affiliations:** ^1^Beijing Key Lab of Physical Fitness Evaluation and Tech Analysis, Capital University of Physical Education and Sports, Beijing, China; ^2^Department of Biomedical Engineering, The Cleveland Clinic, Cleveland, OH 44195, USA; ^3^Beijing Key Laboratory of Learning and Cognition & School of Psychology, Capital Normal University, Beijing, China; ^4^School of Public Health, University of Wisconsin Milwaukee, Milwaukee, WI 53201, USA; ^5^Human Performance and Engineering Research, Kessler Foundation, West Orange, NJ 07052, USA; ^6^Department of Physical Medicine and Rehabilitation, Rutgers New Jersey Medical School, Rutgers University, Newark, NJ 07103, USA

## Abstract

**Background and Purpose:**

Cancer-related fatigue (CRF) is widely recognized as one of the most common symptoms and side effects of cancer and/or its treatment. However, neuropathological mechanisms contributing to CRF are largely unknown, and the lack of knowledge makes CRF difficult to treat. Recent research has shown dissociation between changes in the brain and muscle signals during voluntary motor performance in cancer survivors with CRF, and this dissociation may be caused by an interruption in functional coupling (FC) of the two signals. The goal of this study was to assess the FC between EEG (cortical signal) and EMG (muscular signal) in individuals with CRF and compare the FC with that of healthy controls during a motor task that led to progressive muscle fatigue.

**Method:**

Eight cancer survivors with CRF and nine healthy participants sustained an isometric elbow flexion contraction (at 30% maximal level) until self-perceived exhaustion. The entire duration of the EEG and EMG recordings was divided into the first-half (less-fatigue stage) and second-half (more-fatigue stage) artifact-free epochs without overlapping. The EEG-EMG coupling (measured by coherence of the two signals) in each group and stage was computed. Coherence values at different frequencies were statistically analyzed using a repeated-measure general linear model.

**Results:**

The results demonstrated that compared to healthy controls, CRF participants sustained the contraction for a significantly shorter time and exhibited robust and significantly lower EEG-EMG coherence at the alpha (8~14 Hz) and beta (15~35 Hz) frequency bands. Both the CRF and healthy control groups exhibited significantly decreased EEG-EMG coherence from the less-fatigue to more-fatigue stages at the alpha and beta frequency bands, indicating fatigue-induced weakening of functional corticomuscular coupling.

**Conclusion:**

Impaired functional coupling between the brain and muscle signals could be a consequence of cancer and/or its treatment, and it may be one of the contributing factors to the abnormal feeling of fatigue that caused the early failure of sustaining a prolonged motor task.

## 1. Introduction

Different from the typical feeling of fatigue in everyday life in healthy people, cancer-related fatigue (CRF) experienced by cancer survivors usually during cancer treatment is a persistent subjective sense of tiredness that is not relieved by rest or sleep and may continue for months or even years after treatment is complete. CRF is widely recognized as one of the most common symptoms and side effects of cancer and/or its treatment that occurs in 25% to 99% of people with cancer, particularly in individuals actively undergoing treatment [1–7], while the understanding of its etiology and pathophysiology is very limited. Because of the lack of knowledge of the underlying mechanisms, treatment options for CRF are scarce. CRF has been reported to worsen during motor task exertion and interfere with daily activities [8]. Indeed, cancer survivors with CRF experience muscle weakness and loss of motor endurance that prevent them from performing prolonged motor activities as well as healthy individuals [9].

In a particular study, the authors [9] found that although participants with CRF felt exhausted at the time of failing a sustained muscle contraction, their muscle involved in performing the motor task was not severely fatigued as assessed by physiological measurements. This observation suggests a dissociation between fatigue levels at central (brain) and peripheral (muscle) locations in CRF. Indeed, this dissociation at muscular and supraspinal levels during muscle fatigue is seen even in healthy populations [10–14], but it is significantly exaggerated in individuals with CRF [15–17]. The dissociation between the central and muscular signals with muscle fatigue in healthy and CRF populations seems to suggest an impairment in functional coupling or connectivity between the two signals, and it is interesting to learn if the impairment is more significant in CRF than in the healthy population since CRF patients experience significantly more central than muscle fatigue compared with healthy participants [9]. Distinguishing between the cortical muscular functional coupling pattern in CRF patients with that in healthy controls would help better understand the CRF mechanisms from the neuromuscular perspective and develop effective therapies.

Both cortical and muscular oscillatory activities have been known as common physiological observations. Their coupling of rhythmic oscillations calculated by corticomuscular signal coherence has recently been used to understand cortical control of movement since Conway et al.'s first systematic study based on magnetoencephalography (MEG) and surface electromyography (EMG) signals [18]. There is a general agreement that corticomuscular signal coherence reflects communication between the brain and muscle, which is considered to be related to the control of force and fatigue [19–22] and possibly mediated by the direct corticospinal pathway [23]. Significant correlation between signals of the brain and muscle in the alpha band (8-14 Hz) and beta band (15 to 35 Hz) during voluntary motor actions has been reported in healthy subjects, either in EEG-EMG coherence [24] or MEG-EMG coherence studies [18, 21]. The abnormal features of corticomuscular coherence were also identified in populations with motor disorders, such as stroke [25, 26], tremor [27, 28], and Parkinson's disease [29].

The present study aimed at assessing muscle fatigue-related alterations in functional corticomuscular coupling by measuring EEG-EMG coherence during a sustained submaximal contraction of the elbow flexor muscles in cancer survivors with CRF and compare the outcome with that of healthy controls. It was hypothesized that the functional coupling would be weakened in CRF than in healthy controls due to possible pathophysiological impairment in the central and peripheral nervous systems caused by cancer and/or its treatment [30], and the abnormal corticomuscular signal coupling, among other factors, may worsen fatigue in cancer survivors with CRF.

## 2. Methods

### 2.1. Subjects

Eight right-handed cancer survivors with advanced solid cancer (lung, breast, and gastrointestinal cancer) and CRF (62.9 ± 12.3 years old, 5 men) and 9 right-handed healthy subjects (48.2 ± 14.8 years old, 3 men) participated in the study. The age difference between the two groups was not significant (*P* > 0.05). Among the 8 patients, one (male) had stage 4 breast cancer; two (males), stage 4 colon cancer; one (male), stage 4 kidney cancer; three (two females (both stage 3) and one male (stage 4)), lung cancer; and one (female), stage 4 stomach cancer. Although detailed treatment information of these patients was not clear at the time of the study, it was assured, however, that no patient received chemotherapy or radiation therapy within four weeks prior to the participation in the study and all were postoperative for at least 4 weeks. Eligible patients had a hemoglobin concentration > 10 g/dl and no clinical evidence of polyneuropathy, amyotrophy, or a myasthenic syndrome, by history and physical examination. Significant pulmonary compromise as determined by oxygen dependence was an exclusion criterion for both groups. Patients with weight loss greater than 10% of preillness body weight were excluded. Depressed individuals were identified with a single screening question of “Are you depressed?,” and those with a positive response were excluded from the study [9]. The study was approved by the local Institutional Review Board. All subjects gave informed consent prior to their participation. All subjects were screened by the Brief Fatigue Inventory (BFI) [31] and performed a sustained contraction (SC) of the right-arm elbow flexion at 30% maximal level until self-perceived exhaustion. Elbow flexion force, surface EMG, and high-density EEG were simultaneously recorded during the SC.

### 2.2. Data Recording

#### 2.2.1. Sustained Contraction (SC) to Induce Fatigue

An isometric SC was performed to fatigue the elbow flexor muscles. A target force of 30% maximal voluntary contraction (MVC) force was displayed on an oscilloscope using a horizontal cursor. (The maximal force was measured at the beginning of the experiment.) Participants matched the target with the exerted elbow flexion force in a sitting position with the elbow joint at ~100° and maintained the exerted force on the target until they felt exhausted and were no longer able to continue the contraction. Although motivation for performing the SC was not specifically measured, all participants were verbally vigorously encouraged to continue the SC for as long as possible. The SC was terminated if the exerted force dropped 10% or more for more than 3 s. The forces (maximal and SC) were sensed by a force transducer (JR3 Universal Force-Moment Sensor System, Woodland, CA), acquired by a Spike2 data-acquisition system (1401 Plus, Cambridge Electronic Design Ltd., Cambridge, UK), digitized at 100 samples/s, and stored on the hard disk of a personal computer (PC).

#### 2.2.2. Electromyogram (EMG) Measurements

Bipolar surface EMG was recorded from the belly of the biceps brachii (BB), brachioradialis (BR), and triceps brachii (TB) muscles using Ag-AgCl electrodes (In Vivo Metric, Healdsburg, CA). The recording diameter of each electrode was 8 mm, and center-to-center interelectrode distance was ~3 cm. A reference electrode was placed on the skin overlying the lateral epicondyle near the elbow joint. The EMG signals were amplified (×1000), band-pass filtered (3 Hz–1 KHz), digitized (2000 samples/s), acquired by the Spike2 system, and stored on the hard disk of the PC.

#### 2.2.3. High-Density EEG Measurements

Scalp EEG signals were recorded continuously during the SC using a 128-channel EEG data acquisition system (Electrical Geodesics Inc., Eugene, OR, USA.). All channels of the signals were amplified (×75,000), band-pass filtered (0.1-100 Hz), digitized (250 sample/s), and recorded on the hard disk of a dedicated PC connected to the EEG acquisition hardware and installed with the acquisition and analysis software.

### 2.3. Data Processing and Analysis

The EMG signals were resampled (250 Hz), high-pass filtered at 10 Hz, and rectified. EEG signals were high-pass filtered at 3 Hz. All the EEG data were inspected visually. Recordings with artifacts caused by events such as eye blinks or head movements were excluded, and the corresponding EMG signals were discarded. The entire duration of the EEG and EMG recordings was then divided into the first half (less-fatigue stage) and second half (more-fatigue stage), and subsequently, the signals in each stage were segmented into artifact-free epochs of 256 samples without overlapping (mean = 98.5 epochs, ranged from 44 to 153 for CRF, and mean = 148.5 epochs, ranged from 56 to 264 for controls).

In each stage, a multivariate autoregressive (MVAR) model was applied to each matched epoch of EEG and EMG signals and the coefficients were derived by ARfit MATLAB software [32]. An order of 6 was chosen for the MVAR model based on Schwarz's Bayesian Criterion [33]. Autospectrum and cross-spectrum of the EEG and EMG were calculated from the MVAR coefficients, and the coherence of the two signals was obtained from normalization of the cross-spectrum: *C*_*xy*_^2^(*f*) = |*S*_*xy*_(*f*)|^2^/*S*_*xx*_(*f*)∗*S*_*yy*_(*f*), where *S*_*xx*_(*f*) and *S*_*yy*_(*f*) are the cross-trial smoothed autospectrum of the EEG and EMG signals, *x* and *y*, for a given frequency *f*, and the *S*_*xy*_(*f*) is the cross-trial smoothed cross-spectrum. The frequency resolution was set as 1 Hz. A bootstrap 95% significance level was calculated for every paired EEG-EMG signal at each stage from 100 randomly resampling paired trials [34].

Due to the volume of information, especially the large number of EEG channels, the coherence values of the 128 EEG channels with each of the three muscles (BB, BR, or TB) were grouped into five scalp areas for statistical comparisons: left, right, frontal, central, and parietal [25, 35]. Because no significant EEG-EMG coherence was detected either in the nonfatigue or the fatigue stage at other frequencies, crossing-stage comparisons were limited at the alpha (8-14 Hz) and beta (15-35 Hz) frequency bands. The calculated coherence was normalized by the arc hyperbolic tangent transformation to stabilize the standard deviation [36].

### 2.4. Statistical Analysis

A repeated-measure general linear model was used to statistically compare the coherence between the CRF and control groups at each frequency band by SPSS 12.0 (SPSS Inc., Chicago, IL, USA). The between-subject factor was group and the within-subject factors were fatigue stage, muscle, and scalp area. Additionally, the peak coherence values were also subject to statistical analysis. EMG amplitude of two groups were also compared using a repeated-measure general linear model. Statistical significance level was set at *P* ≤ 0.05. Multiple comparisons were corrected with the Bonferroni method.

## 3. Results

Brief Fatigue Inventory (BFI) scores were higher (*P* < 0.01) in the CRF than in the healthy control group. The mean (±standard deviation) BFI score of the nine questions was 5.2 ± 0.17 for patients and 0.08 ± 0.09 for the controls. Force was well maintained at about 30% of the MVC level, and there was no significant difference of force between stage 1 and stage 2 in both the patient and the control group. However, CRF participants sustained the contraction for a significantly shorter time (335 ± 129 s in CRF vs. 554 ± 140 s in controls, *P* < 0.01), and their MVC elbow flexion force measured before the sustained contraction was significantly lower (187 ± 66 N in CRF vs. 261 ± 75 N, *P* < 0.01) (this means the CRF group sustained a lower absolute force for a shorter time as the target force (30% MVC) was calculated based on the MVC force).

**Figure 1:**
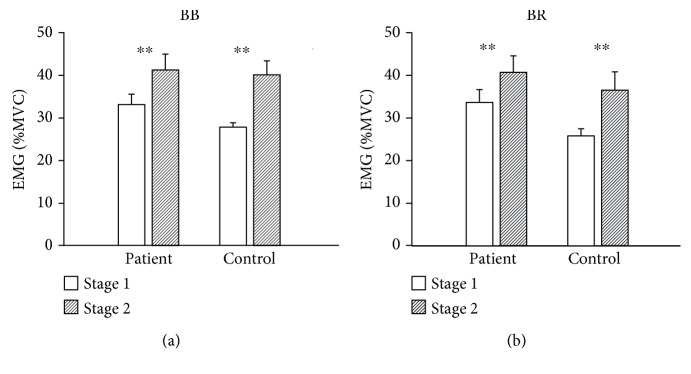
The EMG amplitude of patients and controls in the two stages of the sustained elbow flexion for each agonist muscle. BB: biceps brachii; BR: brachioradialis.

Figure 1 shows the EMG results in the two stages of the sustained elbow flexion of the two groups. The amplitude of surface EMG signals from the elbow flexor muscles (BB, TB) increased significantly (*P* < 0.01) within both groups. No significant differences were found either between groups or different muscles. The increase of the EMG signal of the involved elbow flexor muscles in stage 2 indicated that subjects had to increase their effort to maintain the same force level (by recruiting additional muscle fibers/motor units and/or their activation level) to compensate for the loss of force-generating capability of the fatigued motor units/muscle fibers, which was an indication of muscle fatigue.

**Figure 2:**
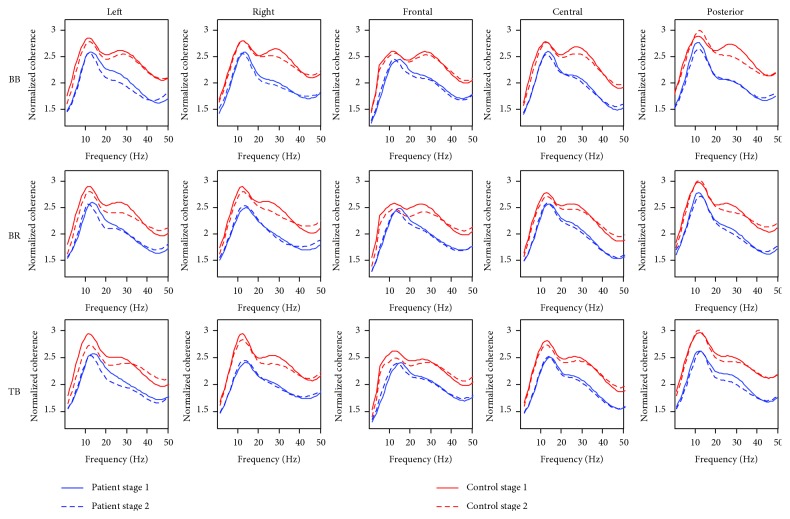
Averaged EEG-EMG coherence spectra related to three muscles in five cortical areas for both patients and controls. BB: biceps brachii; BR: brachioradialis; TB: triceps brachii.

**Figure 3:**
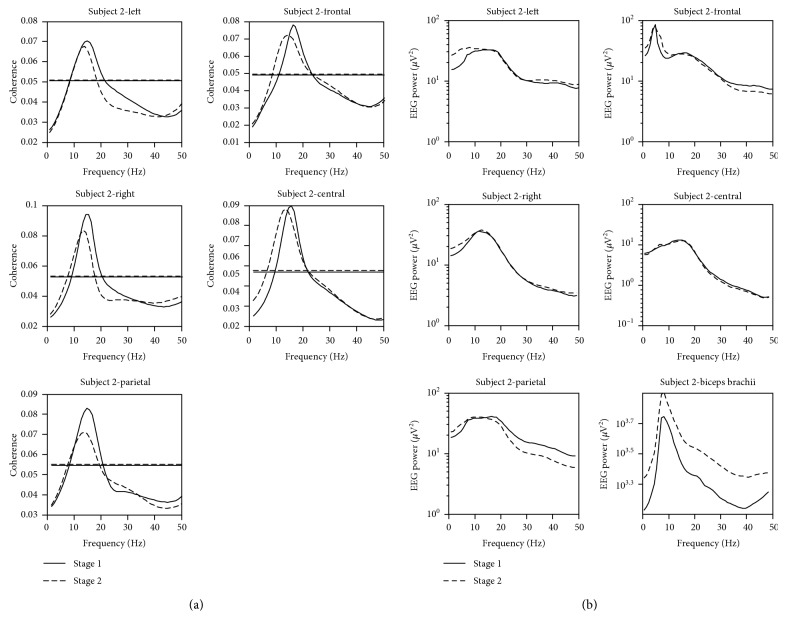
EEG-EMG coherence spectra (a) related to the biceps brachii muscle in five cortical areas and corresponding EEG and EMG power spectra (b) of a typical patient subject data.

EEG-EMG coherence averaged across subjects and electrodes within the cortical area was significantly lower in patients than in controls (Figure 2), especially at the upper beta band (~30 Hz). Control subjects had the first peak coherence value at the upper alpha band (~12 Hz) in both stages of the fatigue process and the second peak value around the upper beta band almost in each muscle and cortical area combination. But CRF patients usually only had the peak coherence value at the upper alpha, and there was an obvious reduction in the value of coherence in the upper beta band in both stages compared to controls. A typical example of EEG and EMG power spectra, EEG-EMG coherence spectra, for one CRF subject is shown in Figure 3.

**Figure 4:**
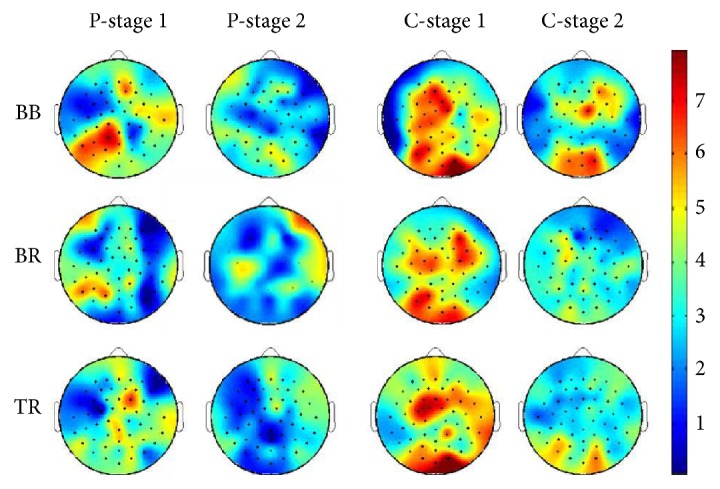
Mapping EEG-EMG coherence based on significant coherence values of the selected 128 EEG channels with EMG of the three muscles at the beta (15-35 Hz) band in CRF patients (left two columns) and healthy subjects (right two columns). The color bar indicates *Z*-transformed coherence values (red means higher and blue lower coherence). The level of coherence declined substantially in stage 2 (fatigue condition, 2nd, and 4th columns) compared with stage 1 (1st and 3rd columns). The coherence values in CRF patients were remarkably lower compared to those in the controls especially in stage 1. BB: biceps brachii; BR: brachioradialis; TB: triceps brachii; P: patients; C: controls.

Figure 4 displays coherence maps (average of the 8 CRF patients and 9 healthy controls) based on the 128 EEG channels with EMG of the three muscles (BB, BR, and TR) for stage 1 (columns 1 (patients) and 3 (controls)) and stage 2 (columns 2 (patients) and 4 (controls)) at the beta band (15-35 Hz). The color bar indicates color-coded Z-transformed coherence values (red color indicates higher coherence value and blue color lower coherence value). The figure shows clearly that (i) the level of coherence declined substantially in stage 2 (more-fatigued condition) compared to stage 1 (less-fatigued condition) in both groups, (ii) the coherence level was higher in the control than in the patient group especially in the baseline stage (stage 1), and (iii) the patterns of the coherence maps between the two groups based on the 128 EEG electrodes and three muscles were dramatically different. Because the EEG sources were not estimated, we could not pinpoint cortical locations whose signal coherence with the EMG was affected by CRF or fatigue. However, by examining the maps in Figure 4, coherence decreased most significantly in the central middle of the frontal lobe and the central posterior areas of the parietal lobe in CRF vs. those in control subjects in stage 1 (compare columns 1 and 3 from left in Figure 4). The fatigue effect on the coherence was most prominent on the left hemisphere in CRF (compare two columns on the left in Figure 4) but almost evenly distrusted on the entire head/brain surface in controls (the two columns on right side of Figure 4).

**Figure 5:**
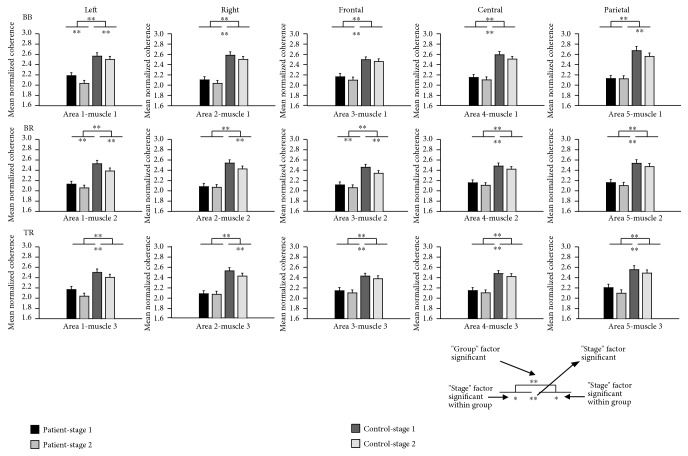
Normalized coherence of all cortical area and muscle combinations in the beta band. The mean coherence was averaged across subjects and electrodes within the cortical area. BB: biceps brachii; BR: brachioradialis; TB: triceps brachii; Left: left cortical area; Right: right cortical area; Frontal: frontal cortical area; Central: central cortical area; Parietal: parietal cortical area. ^∗∗^*P* < 0.01 and ^∗^*P* < 0.05.

**Figure 6:**
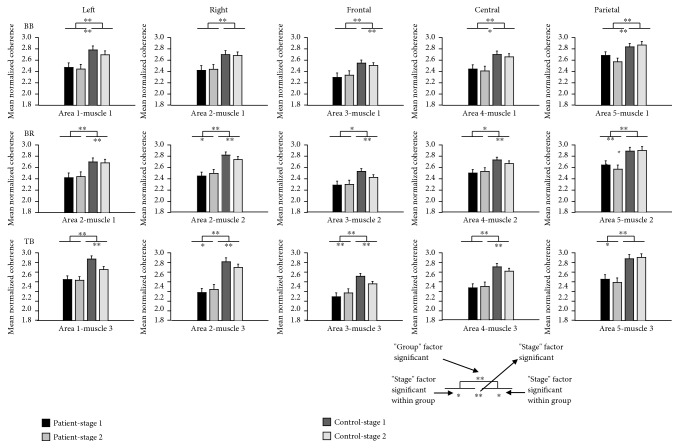
Normalized coherence of all cortical area and muscle combinations in the alpha band. The mean coherence was averaged across subjects and electrodes within the cortical area. BB: biceps brachii; BR: brachioradialis; TB: triceps brachii; Left: left cortical area; Right: right cortical area; Frontal: frontal cortical area; Central: central cortical area; Parietal: parietal cortical area. ^∗∗^*P* < 0.01, ^∗^*P* < 0.05.

The statistical analysis of coherence values by the general linear model of repeated measures showed significantly lower corticomuscular coherence for the CRF group compared with that for the healthy controls at both the alpha and beta bands (beta band: *P* < 0.01, alpha band: *P* < 0.05). The within-subject factor “stage” was significant in both the beta and alpha bands (*P* < 0.01). That means the coherence value decreased significantly in stage 2 compared to stage 1 of the sustained elbow flexion for both the CRF and control groups. The within-subject factor “muscle” was significant in the beta band only (*P* < 0.01). And the within-subject factor “area” was significant in the alpha band only (*P* < 0.01). Since the interactions of the factors were significant at both the alpha and beta bands, further analysis of coherence in each cortical area and muscle combination was necessary. The column chart of normalized coherence of all cortical area and muscle combinations are shown in Figures 5 and 6. At the beta band (Figure 5), the coherence values between the right scalp area (area 2) EEG and EMG of the BR and TB muscles, and the parietal area (area 5) EEG and EMG of the BB muscle were not significantly different between stages 1 and 2 of the motor task in the CRF group, while the differences were significant in the control group. At the alpha band (Figure 6), the difference in the coherence between the two stages in the CRF group was smaller compared to the control group in most of the areas except the parietal cortical area (area 5).

## 4. Discussion

This study, for the first time, showed that functional corticomuscular coupling measured by EEG-EMG coherence was significantly weaker in individuals with CRF compared to healthy controls. And the coupling significantly weakened from less-fatigue to more-fatigue conditions during the sustained elbow flexion contraction in a number of brain areas indicated by signals from multiple EEG electrodes distributed on a large scalp area in both the CRF and control groups.

The novel finding that EEG-EMG coherence was significantly and robustly lower in cancer survivors with CRF suggests significantly impaired functional coupling between the brain and muscular signals in performing a sustained voluntary motor task in individuals with CRF. A voluntary muscle contraction activity is accomplished through generation of a motor command in the brain and transmitting the command signal via the descending pathways to the motor neuron pool in the spinal cord projecting to the target muscle across the neuromuscular junction (NMJ). Since EEG-EMG coherence value reflects the degree of the oscillatory activity “binding” between the central nervous system (CNS) and the muscle [21], any impairment in each component or any block in the pathway during the whole process would increase the dissociation of brain and muscle system signal changes, thus decreasing the corresponding EEG-EMG coherence. Several factors or mechanisms could contribute to the decreased EEG-EMG coherence. One likely candidate is impairment in NMJ transmission. If the central signals cannot be smoothly and efficiently transmitted across the NMJ, the muscle would not be fully recruited into the contraction, which would possibly prevent normal muscle activation and weaken functional coupling between the central and muscular signals. Indeed, a remarkable reduction (~50%) in the NMJ transmission (measured by compound muscle action potential or M-wave elicited by electrically stimulating the motor nerve (pre-NMJ) and recorded on the muscle (post-MNJ)) in cancer survivors with CRF has been reported [9, 37]. Similarly, NMJ propagation efficiency decreased and fatigue increased in prostate cancer patients undergoing radiation therapy, and these symptoms improved 5 to 6 weeks after completion of the radiation intervention [38].

Another factor that could potentially weaken functional corticomuscular coupling during voluntary muscle activation is the diminished central drive from the brain to the muscle. Our previous study has suggested CRF is more centrally mediated fatigue. This was supported by the facts that individuals with CRF exhibited greater subjective fatigue (higher perceived fatigue scores and feeling exhaustion sooner during a prolonged muscle contraction), but physiological indices revealed they experienced less muscle fatigue (compared to healthy controls) at the end of the motor task even though they felt exhausted at the time [15–17]. Voluntary EMG signals at the end the motor task (when participants felt exhausted) suggested diminished central drive to maintain the muscle contraction in CRF participants compared to healthy controls [9, 17], which could be a reason for weakened functional corticomuscular coupling in CRF. With all other factors unchanged, diminished central drive can result in a reduction in the amplitude of muscle force/EMG and perhaps alters frequency content of EEG and EMG signals. These changes could lead to a decrease in the level of corticomuscular coupling or EEG-EMG coherence. Although previous research has indicated that the level of corticomuscular coupling is associated with the magnitude of voluntary force output [21] or central drive, the current study observed an increase in EMG (representing central drive) but a decrease in EEG-EMG coherence in stage 2 of the SC. This observation was contradictory to the positive relationship between voluntary muscle force/EMG and EEG-EMG coherence [39]. Our explanation is that the positive relationship may only hold under nonfatigue conditions. With muscle fatigue in our study, the positive influence of increased central drive on the coherence might have been overridden by effects of fatigue-induced other changes such as frequency content in the EEG and/or EMG signals on the coherence. For example, a frequency band of one signal (e.g., EMG) may have been diminished in stage 2 compared to the other signal (e.g., EEG).

The robust pathophysiological changes in the EEG-EMG coherence in CRF participants observed by the current study may also explain other corticomuscular abnormalities, such as cytokine and neuroendocrine changes in cancer survivors with CRF [6]. Among these changes, the increased proinflammatory cytokines in CRF patients may indicate the switching-on of the immune process by cancer or cancer treatment, which can signal the brain, leading to a variety of effects including fatigue [40]. But exactly how and where these factors take effect are still unknown.

Central and peripheral neuropathy in cancer survivors is well known and thought to contribute to many symptoms such as neuropathic pain, cognitive function impairment, weakness, and fatigue [15, 37, 41, 42]. Both animal and human studies have shown consistent findings of white matter damage in the brain by chemotherapy [43–45]. Numerous studies have also reported peripheral neuropathy caused by chemo drugs [30, 46]. Either the central or the peripheral neuropathy or both are expected to affect generation and conduction of the signals, and communication of the information between the central and peripheral systems. Logically, damage made by chemo or radiation treatment on the central and peripheral systems and its detrimental influence on physiological roles of the systems should interfere with the normal corticomuscular signal coupling for voluntary motor activities.

The significant decrease in EEG-EMG coherence from the less-fatigued to the more-fatigued stage in individuals with CRF was in general consistent with the coherence changes in healthy controls doing the same motor task. This decrease may be due to the inadequate or inhibited drive from various sources that act upon the output neurons [47, 48]. Inhibitory feedback mediated by group III and IV muscle afferents increased along with a decrease in muscle spindle facilitation in progressive muscle fatigue [49–52] or neuromuscular junction propagation changes [53–55]. All these changes are physiologically induced by fatigue motor task, which can be recovered by enough rest or sleep, while those changes that contribute to the lower coherence value in CRF patients compared to the controls in both stages of fatigue motor task may be mainly due to the pathophysiological reasons induced by cancer or cancer treatment, which cannot be recovered just by rest [6, 56]. One interesting observation is that it seems that the coherence value reduction from the less-fatigue to the more-fatigue stage was smaller in CRF than in healthy participants. As can be seen from Figures 5 and 6, in the beta band, the coherence related to the right scalp area EEG and muscle of BR and TB was not significantly different between two stages of the fatigue motor task in the patient group, while it was significant in the control group. And in the alpha band, the difference of two stages' coherence in the CRF group was also smaller compared to that in the control group in most of the areas except the parietal cortical area. An explanation for smaller fatigue-induced EEG-EMG coherence reduction in CRF participants could be that their muscular system was not as fatigued as in the control group [9, 15–17] and perhaps experienced less influence on the coupling of the two signals due to the lower level of muscle fatigue.

Our results suggest that the EEG-EMG coherence of both the CRF patient group and the control group in the beta band was not area dependent (within-subject factor “area” was not significant) but muscular dependent (within-subject factor “muscle” was significant with the *P* value less than 0.01). However, in the alpha band, the coherence in both groups was area dependent (*P* < 0.01) and not muscular dependent. Also, the spatial distribution of the beta band coherence was different from the spatial distribution of the alpha band coherence in patients. The beta band coherence had obvious focus localized around the sensorimotor area while the alpha band coherence had higher value in the parietal cortex. These differences may imply that the mechanisms contributing to the coherence in the alpha and the beta band are at least partially different. It is more likely that the coherence in the beta band is mainly related to the motor control [19–22], while the coherence in the alpha band is more associated with the cognitive component of the motor control [57, 58] besides motor functioning [18, 20]. Cancer survivors with CRF usually also experience cognition-related symptoms, more or less, such as depression [59]. Although we excluded the severely depressed patients in this study by a simple question, cognitive function changes in the participating patients cannot be ruled out.

The study has a number of limitations. First, the sample size was small, which limits our ability to generalize our findings. The major reason for the small sample size was that the study was primarily supported by a small institutional grant with the goal of generating pilot data for future larger-scale studies. Second, the cancer survivors were not limited to a single type of cancer, which made it difficult to explain if a particular cancer contributed more or less to the observed outcomes. Third, although all the participants were verbally encouraged to maintain the sustained contraction for as long as possible, the level of motivation for performing the task was not specifically measured, and therefore, it was possible that one group of participants may have had higher or lower motivation to perform the task than the other, and the difference in motivation might have influenced the motor performance (length of the contraction) as well as the level of corticomuscular coupling. However, given the pilot nature of the study and the robust and significant difference in functional corticomuscular signal coupling during a prolonged voluntary muscle contraction between cancer survivors with CRF and healthy controls, these limitations do not seem to have a significant effect on the major finding of the study.

In conclusion, this study quantified EEG-EMG coherence to evaluate functional corticomuscular coupling in cancer survivors with CRF and healthy controls during a sustained voluntary motor task that led to fatigue. The results indicated significant and robust weakening of corticomuscular signal coupling in CRF compared to healthy controls, which may be caused by central and peripheral neuropathies resulting in cancer treatment and/or the disease itself. Furthermore, both the CRF and healthy participants exhibited decreased functional corticomuscular coupling under muscle fatigue condition with less such decrease in CRF, which is considered to be due to fatigue-induced physiological changes in the sensorimotor system.

## Data Availability

The data used to support the findings of this study are available from the corresponding author upon request.
